# Systemic and local antibiotic prophylaxis in the prevention of *Staphylococcus epidermidis *graft infection

**DOI:** 10.1186/1471-2334-5-91

**Published:** 2005-10-21

**Authors:** Huseyin Turgut, Suzan Sacar, Ilknur Kaleli, Mustafa Sacar, Ibrahim Goksin, Semra Toprak, Ali Asan, Nural Cevahir, Koray Tekin, Ahmet Baltalarli

**Affiliations:** 1 Department of Infectious Diseases and Clinical Microbiology, Pamukkale University, Faculty of Medicine, Denizli, Turkey; 2 Department of Microbiology and Clinical Microbiology, Pamukkale University, Faculty of Medicine, Denizli, Turkey; 3 Department of Cardiovascular Surgery, Pamukkale University, Faculty of Medicine, Denizli, Turkey; 4 Department of General Surgery, Pamukkale University, Faculty of Medicine, Denizli, Turkey

## Abstract

**Background:**

The aim of the study was to investigate the in vivo efficacy of local and systemic antibiotic prophylaxis in the prevention of *Staphylococcus (S.) epidermidis *graft infection in a rat model and to evaluate the bacterial adherence to frequently used prosthetic graft materials.

**Methods:**

Graft infections were established in the subcutaneous tissue of 120 male Wistar rats by implantation of Dacron/ePTFE grafts followed by topical inoculation with 2 × 10^7 ^CFUs of clinical isolate of methicillin-resistant *S. epidermidis*. Each of the graft series included a control group, one contaminated group that did not receive any antibiotic prophylaxis, two contaminated groups that received systemic prophylaxis with teicoplanin or levofloxacin and two contaminated groups that received teicoplanin-soaked or levofloxacin-soaked grafts. The grafts were removed 7 days after implantation and evaluated by quantitative culture.

**Results:**

There was significant bacterial growth inhibition in the groups given systemic or local prophylaxis (*P *< 0.05). Methicillin-resistant *S. epidermidis *had greater affinity to Dacron graft when compared with ePTFE graft in the untreated contaminated groups (*P *< 0.05).

**Conclusion:**

The study demonstrated that the usage of systemic or local prophylaxis and preference of ePTFE graft can be useful in reducing the risk of vascular graft infections caused by staphylococcal strains with high levels of resistance.

## Background

One of the most feared complications of the use of a prosthetic material is the appearance of infection after implant [1-5]. Graft infection often results in prolonged hospitalization, organ failure, amputation and death [4,6,7]. The causative organisms are predominantly *S. auerus *and S. *epidermidis *[4,8]. Most commonly contamination occurs at the time of graft insertion and the most frequent source of infection is from staphylococci from the patient's skin [3,4,9]. The most important strategies for the prevention of prosthetic infection are asepsis and the perioperative administration of systemic antibiotics [10-12]. Moreover, in the case of vascular grafts, alternative methods such as antimicrobials bound in high concentrations to prosthetic grafts have been proposed [2,10,11,13].

However, the antibiotic usage should be guided by local bacterial prevalence and sensitivities, remembering that most infections are caused by staphylococci. Levofloxacin is a fluoroquinolone with an enhanced activity against gram-positive cocci [14,15]. Teicoplanin is a glycopeptide antibiotic and has an excellent bactericidal activity against penicillinase-producing and methicillin-resistant *S. epidermidis *[16,17].

Synthetic vascular prostheses have been developed to supplement the limited supply of native graft materials. But they may act as a foreign body in the patient and may harbour bacteria which results in graft infection [18]. Vascular graft composition and construction have been shown to influence bacterial adherence [3,19].

The aim of the present study was to investigate the in vivo efficacy of local and systemic antibiotic prophylaxis in the prevention of *S. epidermidis *graft infection in a rat model and to evaluate the bacterial adherence to frequently used prosthetic graft materials.

## Methods

This study was carried out in the animal laboratory of our institution. All animals received humane care in compliance with *Principles of Laboratory Animal Care*, formulated by the *Guide for the Care and Use of Laboratory Animals*, prepared by the National Academy of Sciences [20]. This study was also approved by the Pamukkale University Animal Research Ethics Committee, Denizli, Turkey.

The strain of methicillin-resistant *S. epidermidis *used in the present study was isolated from a clinical specimen (graft infection) submitted for routine bacteriological investigation. Identification of the clinical isolate was determined by Gram staining, catalase reaction, tube coagulation test and Api-staph test (biomérieux, Lyon, France). Methicillin sensitivity was investigated by oxacillin disk diffusion test [21]. Levofloxacin and teicoplanin (both from Aventis Pharma) were diluted in accordance with manifacturers' recommendations yielding 1 mg/ml stock solution. Solutions of drugs were made fresh on the day of assay.

One-hundred and twenty adult male Wistar rats (weight range 300 to 350 g) were used in this study. The study included two series composed of 6 groups for each of the woven, gelatin-imregnated polyethyleneterephthalate (Dacron) (Gelwave, Sulzer Vascutek) (D1–6) and expanded polytetrafluoroethylene (ePTFE) (Alpha Graft^®^, Alpha Research) (P1–6) grafts. Each of the series included one group with no graft contamination and no antibiotic prophylaxis (uncontaminated control, Dacron1, ePTFE1), one contaminated group that did not receive any antibiotic prophylaxis (untreated control, Dacron2, ePTFE2), one contaminated group that recieved teicoplanin-soaked grafts (Dacron3, ePTFE3), one contaminated group in which perioperative intraperitoneal prophylaxis with teicoplanin (10 mg/kg) was administered (Dacron4, ePTFE4), one contaminated group that recieved levofloxacin-soked grafts (Dacron5, ePTFE5) and one contaminated group in wich perioperative intraperitoneal prophylaxis with levofloxacin (10 mg/kg) (Dacron6, ePTFE6) was administered. Each rat was anesthetized with a 2:1 mixture of kethamine hydrochloride (100 mg/ml) (Pfizer):xylazine hydrochloride (20 mg/ml) (Bayer) at a dose of 0.75 ml/kg intramuscularly. Rats' hair of the back was shaved and the skin was cleaned with 10% povidone-iodine solution. One subcutaneous pocket was made on each side of the median line by a 1.5 cm incision. Aseptically, 1-cm^2 ^sterile collagen-sealed Dacron or ePTFE grafts were implanted into the pockets. Prior to implantation, in the groups Dacron3, ePTFE3 and Dacron5, ePTFE5 the Dacron and ePTFE graft segments were impregnated with 1 mg/ml teicoplanin and levofloxacin, respectively. Antibiotic bonding was obtained immediately before implantation by soaking grafts for 20 minutes in a sterile solution of antibiotic. The effect of preoperative intraperitoneal teicoplanin and levofloxacin administered 30 minutes before implantation at the standard dose of 10 mg/kg was evalueted in the groups Dacron4, ePTFE4 and Dacron6, ePTFE6, respectively. The pockets were closed by means of skin clips and sterile saline solution (1 ml) containing methicillin-resistant strain *S.epidermidis *at a concentration of 2 × 10^7 ^CFUs/ml was inoculated onto the graft surface by using a tuberculin syringe to create a subcutaneous fluid-filled pocket. The animals were returned to individual cages and thoroughly examined daily. All grafts were removed 7 days following implantation.

The explanted grafts were placed in sterile tubes, washed in sterile saline solution, placed in tubes containing 10 ml of phosphate-buffered saline solution and sonicated for 5 minutes to remove the adherent bacteria from the grafts. Quantitation of viable bacteria was performed by culturing serial dilutions (0.1 ml) of the bacterial suspension on blood agar plates. All plates were incubated at 37°C for 48 hours and evaluated for the presence of the staphylococcal strains. The organisms were quantitated by counting the number of colony-forming units (CFUs) per plate.

Quantitative culture results were presented as arithmetic mean ± standard deviation (S.D.). Differences among the groups were evaluated using one-way analysis of variance (ANOVA), and multiple comparisons between the groups were performed with a posthoc test (Tukey's HSD test). Differences were considered statistically significant when *P *< 0.05. Data were analyzed by a statistical software (SPSS for Windows 11.0; SPSS, Chicago, Illinois).

## Results

None of the animals included in any group died or had clinical evidence of drug related adverse effects, such as local signs of perigraft inflammation, anorexia, vomiting, diarrhoea, and behavioural alterations.

There was no anatomic and microbiological evidence of graft infection in the animals included in the uncontaminated control groups. In contrast, all 20 rats included in the untreated contaminated control groups (Dacron2 and ePTFE2) demonstrated evidence of graft infection, with quantitative culture results showing 3.7 × 10^7 ^± 1.1 × 10^7 ^CFU/ml and 5.3 × 10^6 ^± 2.4 × 10^6 ^CFU/ml, respectively. The quantitative graft cultures of the other groups demonstrated bacterial growth in different counts. The results are summarised in Table 1.

**Table 1:** Quantitative microbiological results of the *in vivo *experiments.

Group^a^	Graft-bonded drug^b^	Intraperitoneal preoperative drug^c^	Quantitative graft culture (CFUs/ml)
Dacron 1^d,e^	--	--	0.0
Dacron 2	--	--	3.7 × 10^7 ^± 1.1 × 10^7^
Dacron 3^d,e^	Teicoplanin	--	5.6 × 10^3 ^± 1.2 × 10^3^
Dacron 4^d,e^	--	Teicoplanin	5.3 × 10^2 ^± 1.2 × 10^2^
Dacron 5^d^	Levofloxacin	--	4.0 × 10^5 ^± 5.5 × 10^4^
Dacron 6^d,e^	--	Levofloxacin	4.9 × 10^4 ^± 4.7 × 10^3^
ePTFE 1^d,e^	--	--	0.0
ePTFE 2^d^	--	--	5.3 × 10^6 ^± 2.4 × 10^6^
ePTFE 3^d,e^	Teicoplanin	--	4.7 × 10^3 ^± 1.2 × 10^3^
ePTFE 4^d,e^	--	Teicoplanin	4.2 × 10^2 ^± 1.4 × 10^2^
ePTFE 5^d^	Levofloxacin	--	3.7 × 10^5 ^± 2.8 × 10^4^
ePTFE 6^d,e^	--	Levofloxacin	4.7 × 10^4 ^± 4.1 × 10^3^

^a ^Each group was performed by 10 animals; Dacron1–6, groups of animals by implantation of Dacron protheses; ePTFE1–6, groups of animals by implantation of ePTFE protheses.

^b ^Graft segments were impregnated with 1 mg/ml teicoplanin; 1 mg/ml levofloxacin.

^c ^Teicoplanin 10 mg/kg, levofloxacin 10 mg/kg.

^d ^Statistically significant when compared with group Dacron2

^e ^Statistically significant when compared with group ePTFE2

The amount of bacterial growth was statistically significantly higher in untreated contaminated Dacron-group (Dacron2) when compared to the other groups (*P *< 0.05). The second highest bacterial growth was observed in untreated contaminated ePTFE-group (ePTFE2) and it was also significantly different from all antibiotic-treated groups, except levofloxacin-bonded Dacron (Dacron5) and ePTFE (ePTFE5) groups (*P *< 0.05). Although the highest reduction in bacterial growth number was observed in the intraperitoneal teicoplanin groups (Dacron4 and ePTFE4), there was no statistically significant difference among the treated contaminated groups (*P *> 0.05).

## Discussion

Various studies have demonstrated that systemic antibiotic prophylaxis reduces the incidence of prosthetic vascular graft infections, but not completely prevent them [22-24]. That's because antibiotic impregnated grafts are arousing interest as they can deliver antibiotic at the time that the graft is at the greatest risk of contamination [22].

In vascular surgery, *S. epidermidis *has been shown to be the leading isolate with infection appearing late after implantation [3,25,26]. So, in order to simulate the clinical setting, we preferred to use an isolate of *S. epidermidis *which was obtained from an infected vascular conduit in our hospital.

Teicoplanin and levofloxacin are attractive options for local and systemic antibiotic prophylaxis in preventing *S. epidermidis *graft infections, because they are effective antibiotics against coagulase positive and negative staphylococci [23,27]. Teicoplanin is used parenterally to treat infections caused by staphylococcal infections since the emergence of methicillin-resistant staphylococci [28]. Recently, teicoplanin has been administered as perioperative antibiotic prophylaxis [29-31]. Teicoplanin has a long half-life and good tissue and bone penetration [32]. In an earlier study at St James's University Hospital, teicoplanin exhibited good penetration into ischaemic tissue, which may be desirable for prophylaxis in vascular surgery [33]. Kester et al. [34] concluded from a two-centre study that a single dose of teicoplanin showed similar efficacy to a three-dose regimen of cephradine plus metronidazole as prophylaxis for wound infection in vascular surgery. It was showed that levofloxacin had greater in vitro and in vivo anti-staphylococcal activity than the other fluoroquinolones such as ofloxacin or ciprofloxacin [35-37]. In previous studies levofloxacin was reported to reach high concentrations in serum and various tissues, following single dose levofloxacin administration [38,39]. In the present study it was found that both local and systemic levofloxacin usage reduced bacterial count in the graft segments when compared to untreated contaminated controls (Dacron2 and ePTFE2) . Although it was not statistically significant, systemic usage of levofloxacin was more effective than the local one. However, interestingly, the reducing efficacy of local usage of levofloxacin on bacterial count was also inadequate to reach a statistical difference when compared to untreated contaminated ePTFE graft (ePTFE2) (*P *> 0.05).

The question remains whether local or systemic antibiotic prophylaxis is the best choice for reducing the risk of prosthetic vascular graft infection. When a local prophylaxis is used, the dose of antibiotic delivered to the operative site could be important in eliminating infections, as most of them are due to contamination at the time of implantation. Simple soaking in an antibiotic solution immediately prior to implantation is an easy way of impregnating the prosthetic graft that can be done extemporaneously by the surgeon himself. But during this procedure the risk of contamination of the graft increases.

Antibiotics having good activity against gram-positive bacteria were used in bonding vascular grafts in experimental models. Hernandez-Richter et al. [40] found that rifampicin and triclosan but not silver was effective in preventing bacterial infection of vascular Dacron graft material. Giacometti et al. [41] confirmed the efficacy of mupirocin-soaked grafts against methicillin-susceptible, methicillin-resistant and vancomycin-intermediate *S. epidemidis*. Ghiselli et al. [42] showed that Temporin A had an antibacterial in vitro activity against methicillin-susceptible and methicillin-resistant *S. epidermidis*. Efficacy of polycationic peptides in preventing vascular graft infection due to methicillin-resistant *S. aureus *with intermediate resistance to glycopeptides was demonstrated to be very good [11]. Dell'Acqua et al. [43] suggested that RNAIII-inhibiting peptide (YSPWTNF-NH2), applied locally and systemically, can completely inhibit drug-resistant *S. aureus *and *S. epidermidis *biofilms. Osada et al. [44] demostrated that levofloxacin incorporated into albumin-sealed Dacron graft had a bactericidal action and adhesive prevention against inoculated *S. aureus *in a graft model.

Soaking of the grafts in solutions of 1 mg/ml of different antibiotics has been shown to be effective in making antibiotic-bonding drugs [45]. Previously, Levofloxacin and teicoplanin, used in 10 mg/kg consantration have been shown to be successful in reducing the risk of prosthetic graft infection [23]. We chosed the same concentrations for the each antibiotic in our model according to these studies' findings.

Our data indicate that, although the difference was not statistically significant, local usage of teicoplanin more effectively reduces the bacterial count in graft segments than the local levofloxacin application and it seems to be the most appropriate antibiotic for local vascular graft prophylaxis. The finding that antibiotic-impregnated grafts alone can not prevent prosthetic vascular graft infection is similar to the results found by other groups [11,22]. The combined usage of systemic antibiotic prophylaxis and drug-bonded grafts has been shown to be more effective in decreasing the incidence of prosthetic vascular graft infections [10,23]. But this combination may have disadvantages like increased intraoperatif contamination risk and cost.

The results of this study demonstrated that both systemic and local prophylactic antibiotic treatment was useful. Although, the difference was not significant, the highest reduction in bacterial number was in the intraperitoneal teicoplanin groups in our study and this finding is parallel with previous studies, [23,37]. Second reason for choosing teicoplanin was its advantage as single dose application. This is certainly more desirable than frequent dosing required by conventional systemic antibiotics or other glycopeptides.

All kinds of prosthetic vascular grafts are susceptible in varying degrees to infection via direct contamination during implantation or bacteremia after operation. Dacron and ePTFE are the most frequently used materials. Surface area and molecular structure differs between the two types of grafts. Graft material of ePTFE is relatively nonporous when it is compared with multifilamented Dacron grafts. EPTFE is more hydrophobic than Dacron, perhaps that's why it is less likely to form bonds with those bacteria in which the cell walls have hydrophobic properties [19,46]. The findings of the present study was similar with previous studies reporting that *S. epidermidis*, *S. aureus *and *Escherichia coli *had greater affinity to Dacron graft when compared with ePTFE [19,47,48]. The bacterial count in the untreated contaminated ePTFE grafts was very low than the untreated contaminated Dacron groups. Untreated contaminated ePTFE graft was found to be almost as effective as levofloxacin-bonded Dacron and ePTFE grafts. Although there were not statistical signifficant differences among the treated contaminated groups, it was observed that bacterial growth was more in Dacron grafts. This finding may be of a clinical importance and may influence the choice of a surgeon when he has to prefer one of these grafts.

Further animal studies are needed to assess the efficacy of commercially available grafts soaked in various antibiotic solutions, against infections after sequental bacterial seeding for up 7 days. Additionally, these results have to be compared with the real situation of an implanted graft in a living human being. It should not be forgotten that antibiotic/antiseptic impregnation is not the only way of protecting synthetic grafts. Modifying the surface characteristics of prosthetic graft to minimise bacterial adherence needs to be investigated further.

## Conclusion

The study demonstrated that the usage of systemic or local prophylaxis and preference of ePTFE graft can be useful in reducing the risk of vascular graft infections caused by staphylococcal strains with high levels of resistance.

## Competing interests

The author(s) declare that they have no competing interests.

## Authors' contributions

HT and SS jointly conceived of the study, secured funding, participated in its design and coordination and co-drafted the manuscript. MS and AB participated in the design and coordination of the study, data analysis and writing the paper. IK, NC and ST participated in the microbiological studies and data analysis. IG and AA participated in the administration of the drugs and in the conduct of the animals' experiments. KT performed the statistical analysis and revised the manuscript. All authors read and approved the final manuscript.

## Pre-publication history

The pre-publication history for this paper can be accessed here:


